# Stress and cancer-related lifestyle factors among African American heterosexual couples

**DOI:** 10.1371/journal.pone.0232577

**Published:** 2020-05-08

**Authors:** Dalnim Cho, Kathrin Milbury, Lorna H. McNeill

**Affiliations:** 1 Department of Health Disparities Research, University of Texas MD Anderson Cancer Center, Houston, Texas, United States of America; 2 Department of Behavioral Science, University of Texas MD Anderson Cancer Center, Houston, Texas, United States of America; University of Colorado Denver, UNITED STATES

## Abstract

Intimate partners can have a profound influence on individuals’ health behaviors. In this exploratory research, we investigated the concordance of cancer-related lifestyle factors including smoking, body mass index, physical activity, fruit and vegetable intake, red meat intake, and alcohol use within African American heterosexual couples. We also examined whether females’ stress is associated with their own (*actor effect*) and males’ cancer-related lifestyle factors (*partner effect*), and vice versa. We analyzed a total of 216 heterosexual couples (i.e., N = 432 individuals) recruited from black churches. Intraclass correlation coefficients (ICCs) were calculated and multilevel modeling in which individuals are nested within couples was conducted. Results showed that there was high concordance of body mass index (ICC = 1.68, *p* < .001), fruit and vegetable intake (ICC = 1.62, *p* < .001), red meat intake (ICC = 1.50, *p* = .001), and alcohol use (ICC = 1.74, *p* < .001) between spouses. A multilevel analysis showed that there were actor and partner effects of stress on females’ BMI; females’ stress was positively associated with their own BMI (actor effect; *β* = .42, *p* = .006) and males’ stress was positively associated with females’ BMI (partner effect; *β* = .39, *p* = .026). Also, females’ stress was positively associated with their own red meat intake (actor effect; *β* = .20, *p* = .019). In conclusion, high concordance of cancer-related lifestyle factors (BMI, fruit and vegetable intake, red meat intake and alcohol use) exists between African American spouses. Given the identified actor and partner effects of stress on females’ BMI, a couple-based lifestyle or weight management intervention that targets both male and female spouses’ stress and coping will be promising, particularly to enhance African American women’s health behaviors. Future studies need to investigate mechanisms underlying concordance and discordance of cancer-related lifestyle factors in African American couples. Also, factors that explain African American male spouses’ health behaviors need to be uncovered.

## Introduction

More than 50% of cancers can be prevented by reducing cancer risk factors such as smoking, obesity, physical inactivity and unhealthy diets, and excessive alcohol use [1]. Yet, African Americans are more likely to be obese [2] physically inactive [3] and eat unhealthily [4] compared to non-Hispanic whites. In addition, African Americans are less likely to succeed in long-term smoking cessation [5] and more likely to report higher rates of alcohol-related illness and injuries than their non-Hispanic white counterparts [6]. Given that African Americans have the highest cancer mortality rates of any racial/ethnic group [7, 8], an important step to advance cancer health equity is to reduce these cancer risk factors among African Americans.

Stress can have substantial, deleterious effects on individuals’ health [9] and it is associated with higher cancer risk factors [10–13]. Within families, research shows that one spouse’s stress may impact the other spouse’s cancer risk factors owing to stress spillover effect [14] implying couples rather than individuals may need to be targeted as a single unit of care or intervention. Accordingly, the aim of the present study is to investigate the concordance of cancer-related lifestyle factors—smoking, body mass index (BMI), physical activity (PA), fruit and vegetable (F&V) intake, red meat intake, and alcohol use—among African American heterosexual couples and to examine the impacts of stress on their own and their spouses’ cancer-related lifestyle factors.

### Concordance of cancer-related lifestyle factors among couples

Significant others can substantially influence one another’s health and health behaviors; physical and mental health are positively associated within couples [15], and married individuals are more likely to be healthy and live longer than non-married individuals [16]. In addition, studies have found that smoking [17], BMI [18], exercise [19], and alcohol use [19, 20] are similar within couples: if a spouse is a smoker, has a high BMI and reports low exercise, and high alcohol use, the other spouse is likely to have similar characteristics.

Several theories of concordance have been proposed, including assortative mating, social control, and shared resources/environment. Assortative mating, which refers to the idea that individuals are more likely to select spouses who are similar to themselves [21], can explain some of the similarities within couples. That is, couples are more likely to show concordance in lifestyle behaviors if their individual behaviors were similar prior to marriage. Social control refers to the direct and indirect attempts from spouses to monitor and shape each other’s behaviors such as telling, reminding, or even threatening spouses in order to promote positive behaviors (e.g., exercise) or to deter negative behaviors (excessive alcohol use) [22]. Finally, the shared resource hypothesis suggests that the characteristics of couple members converge as a result of shared resources such as the physical environment, social networks, and financial resources.

Longitudinal studies have shown that concordance of lifestyle factors within couples can occur during the course of marriage, indicating that concordance may not be explained solely by assortative mating [17, 23–26]. For example, a study that followed 3,889 couples aged 45–65 years for up to 25 years showed that non-obese individuals were more likely to become obese if their spouses became obese [24]. Another study conducted among 5,074 middle-aged and older married couples showed that husbands’ PA trajectories predicted wives’ PA trajectories, and vice versa (i.e., if one partner was ‘stable sedentary’, ‘adopting exercise’, or ‘remaining active’, the other spouse was also likely to be so) [25]. Moreover, a study conducted among 3,722 married and cohabitating couples (age ≥50 years) found that having a spouse who made positive health behavior changes with respect to smoking, PA and weight loss increased the odds of the other spouse making positive changes in these behaviors compared to having a partner who remained unhealthy [17]. Finally, a study among 19,599 married/cohabitating couples and 1,551 future couples that were to marry/cohabitate conducted in Norway showed that couples’ alcohol use can be explained by both assortative mating and convergence over time [27].

However, the concordance of cancer-related lifestyle factors within couples has been mostly overlooked among racial/ethnic minorities. For African Americans, in particular, we could locate one study conducted among 506 African American newly married heterosexual couples. Results showed that husbands’ health and weight management behaviors had a positive association (*r* = .16) with wives’ health and weight management behaviors [28]. However, the study did not assess comprehensive cancer-related lifestyle factors.

### Cross-partner effects of stress on cancer-related lifestyle factors

Stress occurs when an individual perceives that the demands of an external situation (i.e., stressor) exceed the person’s resources for coping [29]. Stress affects individuals’ health through physical, cognitive, and behavioral ways [9]. In particular, stress is related to behavioral changes in that higher stress is associated with higher cancer risk factors such as smoking, inactivity, unhealthy eating (e.g., more fast food consumption, greater energy intake), and alcohol use [10–13]. Furthermore, individuals with greater stress are more likely to have higher BMI and higher odds of being overweight/obese [10, 11].

The impact of stress on cancer-related lifestyle factors may need to be understood in family contexts; one spouse’s stress may impact on the other spouse’s cancer-related lifestyle factors given the stress spillover in marriage, which indicates that stressful experiences in one life domain (external to marriage) can deteriorate relationship functioning (e.g., relationship well-being) [14, 30]. Stress spillover may particularly be important for understanding African Americans’ health given that African Americans are at heightened risk for experiencing stressful events such as financial strain, poverty and racial discrimination [31, 32]. In fact, the above-mentioned study conducted with 506 African American newly married heterosexual couples showed that husbands and wives experiencing financial strain (i.e., stressor) perceived less social support from their spouses [28]. Likewise, research conducted in a prospective study with 346 African American couples showed that greater financial hardship predicted declines in couples’ relationship communication, relationship satisfaction and relationship confidence [33]. These studies are suggestive, but they did not comprehensively include cancer-related lifestyle factors. Also, because they focused on specific types of stressors, the extent to which one spouse’s general stress is related to the other spouse’s cancer-related lifestyle factors remains largely unexplored in African Americans.

### The present study

**Fig 1:**
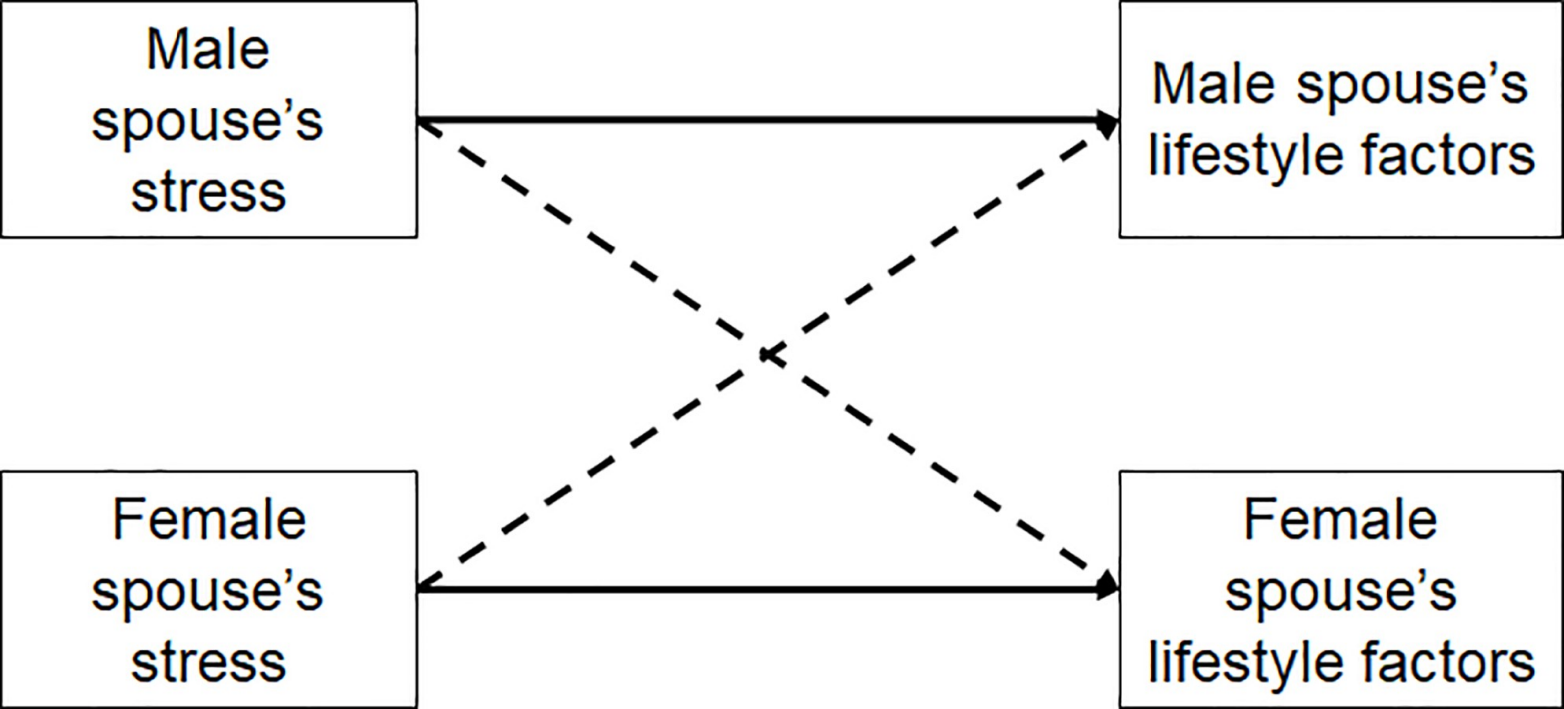
Conceptual framework of the actor-partner interdependence model of the present study. Solid lines reflect actor effects indicating that one spouse’s stress influences his/her own lifestyle factors. Broken lines reflect partner effects indicating that one spouse’s stress influences the other spouse’s lifestyle factors.

The first aim of this exploratory study is to investigate whether cancer-related lifestyle factors—smoking, BMI, PA, F&V intake and red meat intake, and alcohol use—are similar or dissimilar within African American heterosexual couples who live together. We hypothesized that these factors would be positively correlated within couples given the results of studies assessing some of these factors in non-African American couples [17, 24, 25, 27]. The second aim of the current study is to examine whether stress is associated with their own and their spouses’ cancer-related lifestyle factors. That is, we investigated whether females’ stress is associated with their own and males’ cancer-related lifestyle factors, and vice versa. Based on the Actor-Partner Interdependence Model (APIM [34–36]) we hypothesized that a spouse’s stress would be associated with not only his/her cancer-related lifestyle factors (*actor effect*) but also the other spouse’s cancer-related lifestyle factors (*partner effect*) (Fig 1).

## Methods

### Participants and procedures

The participants were analyzed from the Project CHURCH (Creating a Higher Understanding of cancer Research and Community Health) study [37]. Project CHURCH is a community-based participatory research partnering with black churches that aims to investigate psychological, behavioral, social, and environmental factors related to cancer incidence among African American adults.

The participants were recruited from three black churches before and after regular church services, as well as on weekdays. Eligible participants were adults (≥18 years) who were able to read and write in English, lived in the metropolitan Houston area, and had a valid telephone number and home address. Church membership was not required to participate in the study. Recruitment flyers were also distributed during services, and media announcements by ministry were broadcasted during each service to generate significant coverage for the study. Interested participants were screened for eligibility criteria in person at the church. Consent was obtained by research staff. A total of 2,254 African Americans were recruited from 2008 to 2014 from three black churches in Metropolitan Houston, TX areas. Participants received a $30 gift card upon completion of a survey. This study was approved by the Institutional Research Board at the University of Texas MD Anderson Cancer Center.

As Project CHURCH was not specifically designed for a couples study, we implemented the following strategies in order to identify couples. First, based on a survey question asking marital status, we identified 996 participants who were married (*n* = 942) or living together with a partner (*n* = 54). Second, we reviewed self-reported residential addresses and determined individuals who shared the same address. We excluded 564 participants as: 1) they did not have an individual with the matched address (i.e., they were married/partnered, but the partner did not participate in the study; *n* = 516); 2) the marital status of the individual having the matched address was neither married nor cohabitating (i.e., the cohabitating individual was not an intimate partner; *n* = 45); and 3) there were discrepancies in reported addresses and thus we could not confirm couple status (*n* = 3). Thus, a total of 216 heterosexual couple dyads (i.e., 432 individuals) were selected and analyzed.

### Measures

#### Stress

Perceived stress was assessed with the 4-item Perceived Stress Scale [38]. Participants were asked to respond the extent to which they felt or thought to each item during the last month (e.g., “In the last month, how often have you felt that you were unable to control the important things in your life?”; “In the last month, how often have you felt difficulties were piling up so high that you could not overcome them?”) from 1 (*never*) to 5 (*very often*).

#### Cancer-related lifestyle factors

Six cancer-related lifestyle factors (Smoking, BMI, PA, F&V intake, red meat intake, and alcohol use) were assessed. First, in order to assess current smoking status, two items were used: “Have you smoked at least 100 cigarettes in your entire life?” (*yes/no*) and “Do you now smoke cigarettes every day, some days, or not at all?” (*every day*, *some days*, *not at all*). Current smokers indicate individuals who have smoked at least 100 cigarettes and are currently smoking every day or some days. Second, weight and height were objectively assessed by research staff on site and BMI was calculated (weight in kilograms divided by height in meters squared). Third, PA was assessed with the 6-item International Physical Activity Questionnaire-Short Form (IPAQ-SF), which is a widely used PA instrument [39] that produces MET (metabolic equivalent) minutes of walking and moderate and vigorous PA assessment within the past 7 days. Fourth, daily servings of F&V were assessed using the Dietary Intake Questionnaire [40]. Fifth, red meat intake was assessed with four items asking how often participants consumed meat products within the past month (e.g., “How often have you eaten beef, ham, pork, or lamb in a sandwich or in a mixed dish, for example, in a stew, casserole, or lasagna?” Participants were asked to respond from 1 (*never*) to 6 (*once or more a day*) and weekly servings of red meat consumption were produced. Finally, a sum for weekly alcohol use was calculated by a series of questions asking about any alcohol consumption during the past year (*yes/no*) and the total number of drinks consumed on average for each day of the week (e.g., “How many drinks do you consume on an average [Monday]?”). One drink was defined as one beer, one wine cooler, one glass of wine, one shot, or one mixed drink.

### Statistical analysis

First, we conducted paired t test (two-tailed) for PA, FV, red meat, and alcohol use and Signed-rank test for smoking to examine differences across each cancer-related lifestyle factor between male and female partners. Second, Intraclass Correlation Coefficient (ICC) between partners was calculated. ICC of dyadic data estimates the relationship in the outcome variable between the members within the same dyad, and thus, provides the measures of non-independence within couples in our data. It is calculated as the proportion of between-dyad variance to the total variance in the outcome variable, which can be explained by the dyadic clustering. We used the following criteria for the magnitude of ICC [41]: .01 (small); .10 (medium); .25 (large). In addition, correlational analyses were conducted to investigate associations between variables. Specifically, a bivariate correlational analysis (for PA, FV, red meat, and alcohol use) or a simple logistic analysis (for smoking) was conducted to investigate associations between one couple member’s stress and the other couple member’s cancer-related lifestyle factors. Finally, we conducted an analysis of the APIM [34–36] in multilevel modeling framework, in which individuals are nested within couple dyads. Specifically, actor's stress and partner's stress as main predictors, gender (female = 0, male = 1) as the distinguishing variable, and the interaction terms between stress and gender to differentiate actor's and partner's effects were regressed onto each cancer-related lifestyle factor. The multilevel analysis was conducted only for the cancer-related lifestyle factors that had significant ICC at *p* < .05 level between male and female partners. Stata (Stata Statistical Software: Release 14. College Station, TX) was used for the data analysis [42].

## Results

### Differences within couples in stress and cancer-related lifestyle factors

On average, the sample was comprised of middle-aged people: mean age for male spouses was 51.27 and mean age for female spouses was 48.69 years (Table 1). About 60% of female spouses reported having at least a college degree compared to about 50% of male spouses. Mean stress was 4.17 for male spouses and 4.62 for female spouses. Majority of the participants (92.99%) were non-smokers; only a total of 30 individuals (28 couples at least one dyadic member is a smoker) reported that they smoke everyday (*n* = 16) or some days (*n* = 14). Mean BMI was 31.08 for male spouses and 31.59 for females. Both spouses did not meet the recommended level of F&V intake (i.e., at least 5 servings/day) on average. However, their level of alcohol use was far below the threshold for moderate drinking (i.e., up to 14 drinks per week for men and up to 7 drinks for women).

**Table 1:** Demographics, stress and cancer-related lifestyle factors of the study sample.

Variable	Male partner (Husband) n = 216		Female partner (Wife) n = 216		Paired t (2-tailed)
	*M* or %	*SD*	*M* or %	*SD*	
**Age, years**	51.27	12.36	48.69	11.59	7.59*** (215)
**Education (at least college)**	48.15%		58.80%		47.28***(1)
**Stress**	4.17	2.81	4.62	3.26	-1.76 (212)
**Current smoking**^**a**^	22 (10.28%)		8 (3.74%)		
**BMI**	31.08	6.58	31.59	7.54	-.86 (213)
**Vigorous PA (per week)**	2223.45	2516.65	1199.28	1806.06	4.96*** (187)
**Moderate PA (per week)**	993.07	1220.59	646.91	891.24	3.00** (189)
**Walking (per week)**	1300.53	1385.84	1078.98	1250.15	1.70 (190)
**F&V intake (serving/per day)**	2.86	2.20	3.47	2.41	-3.14** (215)
**Red meat intake (serving/week)**	5.30	3.85	4.67	4.08	1.87 (215)
**Alcohol use (per week)**	2.67	4.52	1.84	3.44	2.52* (212)

BMI = Body mass index; PA = Physical activity; F&V = Fruit and vegetable; M = Mean; SD = Standard deviation.

^a^Results indicate frequency (n and %)

*p < .05

**p < .01

***p < .001.

Table 1 also presents differences in stress and cancer-related lifestyle factors within couples. Results showed that there were no significant differences with respect to stress (*t*[212] = -1.76, *p* = .080), BMI (*t*[213] = -.86, *p*>.05), walking (*t*[190] = 1.70, *p*>.05), and red meat intake (*t*[215] = 1.87, *p*>.05) within couples. However, significant differences were found with regard to current smoking (*p* = .009), moderate (*t*[189] = 3.00, *p* < .01) and vigorous PA (*t*[187] = 4.96, *p* < .001), F&V intake (*t*[215] = -3.14, *p* < .01), and alcohol use (*t*[212]2.52, *p* < .05). In general, male spouses engaged in more moderate and vigorous PA, ate less F&V, and drank more alcohol than their female spouses.

### Concordance in cancer-related lifestyle factors within couples

The within-person correlation analyses between stress and cancer-related lifestyle factors and ICC are presented in Table 2. ICC of smoking was .07, which was not statistically significant (*p* = .155). Of the 28 couples in which at least one couple member is a smoker, only two couples were concordant in smoking status (i.e., both were current smokers). ICCs of stress and continuous lifestyle factors were: .25 for stress (*p* < .001); .25 for BMI (*p* < .001); .11 for vigorous PA (*p* = .073); .00 for moderate PA (*p* = .964); .06 for walking (*p* = .196); .24 for F&V intake (*p* < .001); .20 for red meat intake (*p* = .001); and .27 for alcohol use (*p* < .001).

**Table 2:** Correlations between stress and cancer-related lifestyle factors for male and female spouses.

Variable	Stress	Smoking^a^	BMI	Vigorous PA	Moderate PA	Walking	F&V	Red meat	Alcohol use
**Stress**	.25***	1.13	.05	.11	.15*	.09	-.02	.08	.12
**Smoking**^**a**^	.97	.07	.93	1.00	1.00	1.00	1.02	1.03	1.10*
**BMI**	.22**	.95	.25***	.05	.01	.10	-.02	.05	-.02
**Vigorous PA**	-.04	1.00	-.05	.11	.38***	.19**	.14*	-.13	.18*
**Moderate PA**	-.16	1.00	.07	.52***	.00	.32***	.03	-.01	.08
**Walking**	-.09	1.00	.00	.35***	.48***	.06	.07	-.04	-.01
**F&V**	-.01	1.14	.10	.07	.10	.14*	24***	-.07	-.05
**Red meat**	.19**	1.18*	.10	-.16*	-.07	-.01	-.01	.20**	.14*
**Alcohol use**	.09	1.21**	-.07	-.00	.02	.09	.11	.13	.27***

BMI = Body mass index; PA = Physical activity; F&V = Fruit and vegetable. Female spouse (wife) correlates are on lower diagonal and shaded in light grey, male spouse (husband) correlations on upper diagonal, and intraclass correlation coefficient (ICC) are on the diagonal and shaded in dark grey.

^a^Numbers indicate odds ratio.

*p < .05

**p < .01

***p < .001.

### Multilevel analysis

Results of the APIM are shown in Table 3. With regard to BMI, there was a main effect of gender in that male spouses had higher BMI (*β* = 2.70, *p* = .026). Also, there were actor and partner effects of stress on BMI; female spouses’ BMI was positively associated with their own stress (actor effect; *β* = .42, *p* = .006) and male spouses’ stress (partner effect; *β* = .39, *p* = .026). However, male spouses’ BMI was not significantly associated with their own stress (*β* = -.03, *p* = .205) and female spouses’ stress was not significantly associated with male spouses’ BMI (*β* = -.43, *p* = .074).

**Table 3:** Actor-partner effects on cancer-related lifestyle factors.

Outcome	Predictor	*β* (SE)	95% CI
**BMI**	Gender	2.70* (1.21)	.33, 5.08
	Actor stress^a^	.42** (1.53)	.12, .72
	Partner stress^b^	.39* (.18)	.05, .74
	Gender × Actor stress^c^	-.30 (.24)	-.78, .17
	Gender × Partner stress^d^	-.43 (.24)	-.90, .04
**F&V intake**	Gender	-.57 (.40)	-1.35, .22
	Actor stress^a^	.00 (.05)	-.10, .10
	Partner stress^b^	-.04 (.06)	-.15, .08
	Gender × Actor stress^c^	.00 (.08)	-.15, .16
	Gender × Partner stress^d^	-.01 (.08)	-.17, .14
**Red meat intake**	Gender	1.50* (.72)	.10, 2.90
	Actor stress^a^	.20* (.20)	.03, .37
	Partner stress^b^	.16 (.10)	-.03, .36
	Gender × Actor stress^c^	-.12 (.13)	-.38, .14
	Gender × Partner stress^d^	-.08 (.13)	-.34, .18
**Alcohol use**	Gender	.61 (.69)	-.75, 1.96
	Actor stress^a^	.05 (.09)	-.12, .22
	Partner stress^b^	.18 (.10)	-.02, .38
	Gender × Actor stress^c^	.10 (.14)	-.17, .37
	Gender × Partner stress^d^	-.05 (.14)	-.32, .22

SE = Standard error; BMI = Body mass index; PA = Physical activity; F&V = Fruit and vegetable. Gender: 0 = female and 1 = male.

^a^actor effect of female spouse’s stress on her own cancer-related lifestyle factor.

^b^partner effect of male spouse’s stress on female spouse’s cancer-related lifestyle factor.

^c^actor effect of male spouse’s stress on his own cancer-related lifestyle factor.

^d^partner effect of female spouse’s stress on male spouse’s cancer-related lifestyle factor.

*p < .05

**p < .01.

With respect to red meat intake, there was a main effect of gender showing that male spouses reported higher consumption of red meat (*β* = 1.50, *p* = .036). Female spouses’ stress was positively associated with her own red meat consumption (actor effect; *β* = .20, *p* = .019); however, male spouses’ stress was not significantly associated with their own red meat intake (actor effect; *β* = -.12, *p* = .379). There was no partner effect on read meat intake. That is, male spouses’ stress was not significantly associated with female spouses’ red meat consumption (partner effect; *β* = .16, *p* = .094) and female spouses’ stress was not significantly associated with male spouses’ red meat consumption (partner effect; *β* = -.08, *p* = .552). Neither actor nor partner effects of stress on F&V intake and alcohol use was found.

## Discussion

In this exploratory research, we investigated concordance of cancer-related lifestyle factors—smoking, BMI, PA, F&V intake, red meat intake and alcohol use—within African American heterosexual couples. We also sought to examine whether stress is associated with their own (actor effect) and their spouses’ cancer-related lifestyle factors (partner effect).

Results showed that cancer-related lifestyle factors including BMI, F&V intake, red meat intake, and alcohol use were highly concordant in African American couples. That is, if one spouse had higher BMI, F&V intake, red meat intake and alcohol use, the other spouse was more likely to do so. Particularly, the high concordance of F&V intake and red meat intake may be because female spouses typically prepare family meals [43].

Surprisingly, however, smoking and PA were not concordant between the spouses. The non-significant concordance in smoking may be partly due to the gender difference in that men’s smoking rates are 20.9%, which is the highest among all racial/ethnic groups in US men, whereas women’s smoking rates are 13.5% [44]. However, it should be also noted that the majority of the participants in the present study were non-smokers, perhaps due to the fact that this study was conducted among a faith-based sample. The non-significant concordance between spouses’ PA was unexpected as well. We reason that this result likely reflects the multiple circumstances in which PA can occur. For example, walking takes place in many different situations and the assessment of walking activities included walking at work and home, walking to travel from place to place, and any other walking done for recreation, sport, exercise, or leisure.

Multilevel analyses showed that stress might be a risk component for cancer-related factors, particularly among African American female spouses (rather than male spouses); specifically, there was an actor effect of stress on female spouses’ BMI and red meat intake. That is, female spouses’ higher stress was associated with their own higher BMI and higher red meat intake. Furthermore, there was a partner effect of stress on female spouses’ BMI in that male spouses’ higher stress was associated with female spouses’ higher BMI. Thus, to enhance African American women’s health not only her own but also her spouse’s stress may need to be addressed. For example, a couple-based weight management intervention or lifestyle intervention that includes stress and coping among African Americans may be developed to reduce African American women’s BMI. Given that African American women have the highest obesity rate (54.8%) than other racial/ethnic groups in the US [45], this couple-based intervention will be particularly promising to enhance health of African American women who have significant others.

However, there were no actor effects of stress on males’ BMI and red meat intake. In addition, there was no partner effect of stress on male spouses’ BMI. That is, an African American man’s cancer-related lifestyle factors were not explained by his own or his spouse’s stress level. These results may indicate gender differences in stress and coping: Overall, it is possible that female spouses’ health behaviors may be more vulnerable to stress than male spouses’ [46]. It is also possible that male spouses may cope with their own or their spouses’ (spilled over) stress in ways that are not necessarily health behavior debilitating. In fact, stress was significantly positively correlated with male spouses’ own moderate PA (*r* = .15) and although it was not significant, the association trend with stress and other PAs was positive as well (*r* = .11, for vigorous PA and *r* = .09 for walking). In contrast, while not significant, the association trend between stress and PA was negative in female partners (*r* = -.04 for vigorous PA; *r* = -.16 for moderate PA; and *r* = -.09 for walking). We reason that these results may indicate that male spouses may cope with stress by engaging in PA. Future research is highly warranted regarding African American male spouses’ stress, coping, and cancer-related lifestyle factors.

However, note that there were no actor and partner effects of stress on other cancer-related lifestyle factors. We reasoned that the non-significant results may be partly explained by the contents and assessment of stress in the present study. We measured *overall* stress, but specific stress (e.g., health-related stress, residential-related stress, work-related stress) may exert a stronger impact on cancer-related lifestyle factors. Furthermore, we assessed global, aggregated stress at the between-person level, but there is within-person variability in stress [47] and the stress-behavior link at the between-person level does not necessarily inform and reflect that at the within-person level [48].

The present study has several limitations. First, the original study was not specifically designed to recruit and examine couples. Thus, couple status had to be selected based on several criteria (marital status, gender, and age) and potential covariates (marital quality, relationship duration) could not be controlled. It is possible that couples who have higher marital quality particularly mutuality or longer relationship length may have higher concordance of cancer-related lifestyle factors; future studies need to examine whether the results are moderated by these relationship characteristics. Second, the present study did not address concordance theories, and in fact, our cross-sectional analyses cannot completely rule out assortative mating as a determining factor. The results should be confirmed by longitudinal and prospective studies. Third, except for BMI, which was calculated based on objectively measured weight and height, other lifestyle factors were assessed with self-report measures which may be susceptible to error and biases. Finally, this study was conducted among a faith-based sample and our sample comprised mostly middle-aged couples, had relatively high level of education, and only had heterosexual couples. Compared with other studies, the level of stress [49] and risky behaviors such as smoking and alcohol use [50] were lower in our faith-based sample; participants probably cope with stress well through their religion and refrain from those risky behaviors which may not be favorably evaluated by their religion. Thus, the generalizability of our results to African Americans who do not go to church may be limited.

## Conclusions

Nevertheless, this is one of the very few couple-based studies specifically focusing on African Americans. We advance the existing literature by examining diverse cancer-related lifestyle factors and investigating the actor and partner effects of stress on cancer-related lifestyle factors. Our results support the possibility of providing couple-focused weight management interventions to potentially reduce cancer risk in partnered African American women. Future studies need to investigate mechanisms of how spouses influence one another and understand the complex and nuanced couple dynamics among African Americans. In fact, the current body of research does not yet support that interventions targeting couples are more effective than those targeting individuals [51, 52]. Therefore, much research needs to be performed to better understand concordance and discordance of cancer-related lifestyle factors for African American couples, to examine factors explaining the partner effects, and to investigate mechanisms of partner effects to enable the development of effective lifestyle and weight management interventions. In addition, uncovering factors that influence African American male health is an important future endeavor, given their lowest longevity in the US [53]. Such studies hold promise for lowering health behaviors associated with increased risk of cancer, thus enhancing cancer prevention and lessening the burden of cancer in traditionally underserved and overlooked populations.
